# Comparative performance of cardiovascular risk prediction models in people living with HIV

**DOI:** 10.4102/sajhivmed.v23i1.1395

**Published:** 2022-11-15

**Authors:** Irtiza S. Tahir, Alinda G. Vos, Johanna A.A. Damen, Roos E. Barth, Hugo A. Tempelman, Diederick E. Grobbee, Karine Scheuermaier, Willem D.F. Venter, Kerstin Klipstein-Grobusch

**Affiliations:** 1 Julius Global Health, Julius Center for Health Sciences and Primary Care, University Medical Center Utrecht, Utrecht, the Netherlands; 2 Ezintsha, Faculty of Health Sciences, University of the Witwatersrand, Johannesburg, South Africa; 3 Julius Center for Health Sciences and Primary Care, University Medical Center Utrecht, Utrecht, the Netherlands; 4 Department of Infectious Diseases, University Medical Center Utrecht, Utrecht, the Netherlands; 5 Ndolvu Care Group, Groblersdal, South Africa; 6 School of Physiology, Faculty of Health Sciences, University of the Witwatersrand, Johannesburg, South Africa; 7 Division of Epidemiology and Biostatistics, School of Public Health, Faculty of Health Sciences, University of the Witwatersrand, Johannesburg, South Africa

**Keywords:** cardiovascular disease risk, Framingham risk score, D:A:D risk score, Atherosclerotic Cardiovascular Disease Risk Score, people living with HIV, sub-Saharan Africa

## Abstract

**Background:**

Current cardiovascular risk assessment in people living with HIV is based on general risk assessment tools; however, whether these tools can be applied in sub-Saharan African populations has been questioned.

**Objectives:**

The study aimed to assess cardiovascular risk classification of common cardiovascular disease (CVD) risk prediction models compared to the Data Collection on Adverse Events of Anti-HIV Drugs (D:A:D) 2010 and 2016 models in people living with HIV.

**Method:**

Cardiovascular disease risk was estimated by Framingham Cardiovascular and Heart Disease (FHS-CVD, FHS-CHD), Atherosclerotic Cardiovascular Disease (ASCVD) and D:A:D 2010 and 2016 risk prediction models for HIV-infected participants of the Ndlovu Cohort Study, Limpopo, rural South Africa. Participants were classified to be at low (< 10%), moderate (10% – 20%), or high-risk (> 20%) of CVD within 10 years for general CVD and five years for D:A:D models. Kappa statistics were used to determine agreement between CVD risk prediction models. Subgroup analysis was performed according to age.

**Results:**

The analysis comprised 735 HIV-infected individuals, predominantly women (56.7%), average age 43.9 (8.8) years. The median predicted CVD risk for D:A:D 2010 and FHS-CVD was 4% and for ASCVD and FHS-CHD models, 3%. For the D:A:D 2016 risk prediction model, the figure was 5%. High 10-year CVD risk was predicted for 2.9%, 0.5%, 0.7%, 3.1% and 6.6% of the study participants by FHS-CVD, FHS-CHD, ASCVD, and D:A:D 2010 and 2016. Kappa statistics ranged from 0.34 for ASCVD to 0.60 for FHS-CVD as compared to the D:A:D 2010 risk prediction model.

**Conclusion:**

Overall, predicted CVD risk is low in this population. Compared to D:A:D 2010, CVD risk estimated by the FHS-CVD model showed similar overall results for risk classification. With the exception of the D:A:D model, all other risk prediction models classified fewer people to be at high estimated CVD risk. Prospective studies are needed to develop and validate CVD risk algorithms in people living with HIV in sub-Saharan Africa.

## Introduction

Sub-Saharan Africa (SSA) has the largest HIV-infected population in the world comprising around 70% of the global HIV population of 37.7 million.^1^ While antiretroviral therapy (ART) has reduced morbidity and mortality in people living with HIV (PLHIV),^2^ complications associated with ageing such as cardiovascular disease (CVD), cancer, osteoporosis, and other end-organ diseases are increasing.^3,4^ Cerebrovascular disease and CVD were ranked as the fourth and fifth leading causes of death in Africa in 2015.^5^

In PLHIV early onset of CVD has been observed.^6,7,8,9^ Risk of CVD is increased up to two times in PLHIV.^10^ It is likely that inflammation from HIV infection, which only partially resolves on ART contributes to CVD development. Moreover, there is an association between HIV and traditional CVD risk factors,^11,12^ and adverse reactions of the ART medication may also play a role^13^.

To identify PLHIV at high-risk of CVD, the Data Collection on Adverse Events of Anti-HIV Drugs (D:A:D)^14^ risk algorithm can be used. However, the D:A:D prediction algorithm was developed based on data from a predominantly high-risk male population residing in high-income countries and did not include clinical outcomes such as cardiac failure, transient ischemic attack (TIA) and peripheral artery disease (PAD). Therefore, its application for CVD risk prediction in PLHIV in SSA has been questioned. Currently, there is no validated CVD risk prediction tool for PLHIV in low- and middle-income settings.^15,16,17^

We compare CVD risk estimation by common CVD risk prediction models. Reported angina, heart failure, or heart attack by a parent or sibling before the age of 60 years was considered as family history of CVD (the Framingham Heart Study-Cardiovascular Disease [FHS-CVD],^18^ the Framingham Heart Study-Coronary Heart Disease [FHS-CHD],^19^ the Atherosclerotic Cardiovascular Disease Risk Score [ASCVD]^20^ and D:A:D 2016 relative) with the D:A:D 2010 model^14,21^ in PLHIV in rural South Africa.

## Methods

### Study design and study population

This study was conducted using baseline data from the Ndlovu Cohort Study (NCS),^21^ a which is investigating the association of HIV infection, HIV treatment, and conventional CVD risk factors on CVD risk in rural South Africa.^8,22^

Baseline assessment of study participants of the NCS was undertaken between November 2014 and August 2017 in Elandsdoorn, Limpopo, South Africa. Overall, 1927 people (male and female) participated in the study, among them 887 infected with HIV. Participants for the NCS were recruited through community campaigns, at local events and shopping centres, as well as at the Ndlovu Medical Centre (NMC). The NMC included a large rural HIV treatment facility, contracted by the South African Department of Health, providing free-of-charge HIV treatment and follow-up to ≈3700 HIV-positive patients. Details on the design and the methods of the NCS have been described previously.^21^

The current analysis is based on 735 HIV-infected participants aged 30 to 75 years. The age cut-off points were chosen according to the age range for both Framingham and ASCVD prediction models. Established CVD was defined as self-reported angina pectoris, stroke, myocardial infarction and heart failure. Participants with established CVD were excluded from the current sub-study. Figure 1 provides details on the inclusion and exclusion criteria.

**FIGURE 1:**
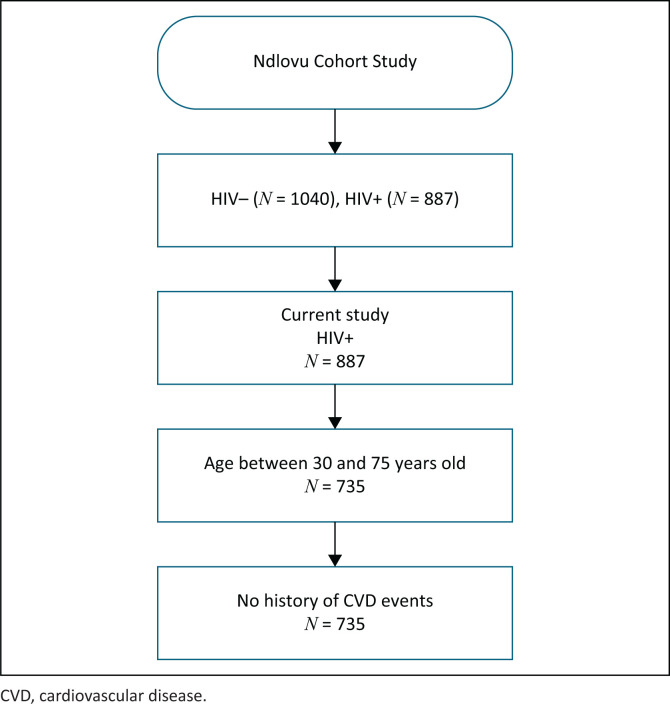
Flow chart of study.

### Data collection

#### General characteristics

Information on age, sex, income, civil status, demographics, socio-economic status (SES), medical history, and medication use (both HIV-related and for other medical issues), family history, and lifestyle were collected by use of standardised questionnaires.^23^ Detailed information on past and current HIV treatment was recorded including the time between diagnosis and treatment initiation, medication prescription and treatment response (latest plasma HIV-1 viraemia, latest CD4+ cells). ART status was assessed by self-reporting and complemented with information from an electronic HIV registry (TIER.net).^24^ Physical activity was evaluated by use of the international physical activity questionnaire (IPAQ).^25^ This questionnaire assesses the intensity of physical activity and sitting time in order to estimate total physical activity in metabolic equivalents (MET)-min/week. Information was collected on smoking and alcohol status (i.e. ever or never use of cigarettes or other tobacco products, and alcoholic beverages, and the participants’ current use of cigarettes and alcoholic beverages). History of raised blood pressure, any drugs taken for blood pressure (previously or currently), history of diabetes, and other CVD risks were also assessed. Participants with a body mass index (BMI) > 30 kg/m^2^ were considered obese. Diabetes mellitus (DM) was defined based on haemoglobin A1c (HbA1c) > 6.5 mmol/L or being on diabetes medication. Income in this low-resource setting was categorised as low (< R648.00/month [South African rand]), medium (R648.00 – R992.00/month) and high (> R992.00/month).^26^ Reported angina, heart failure, or heart attack by a parent or sibling before the age of 60 years was considered as family history of CVD.

#### HIV testing procedure

Participants underwent HIV testing unless their status was known to be HIV-positive and on ART. Testing was performed with an antibody-based point-of-care test (Advanced Quality Rapid HIV Test, InTec Products, Xiamen, China). Participants that tested positive were retested with a second point-of-care test (ABON HIV 1/2/0 Trilines HIV Rapid Test Device, ABON Biopharma, Hangzhou, China).^27^ Participants who tested positive for HIV upon enrolment in our study were referred to the NMC, or any other local HIV treatment facility, to initiate ART. Participants with confirmed HIV-positive status were recruited from the NMC or outreach testing programmes. Documentation of a positive HIV test result was needed for all patients to be eligible for enrolment as HIV-infected participants in the study.

#### Physical measurements

Anthropometric measurements included weight, height, and waist and hip circumference. Blood pressure was measured in all participants in a seated position after 5 min of rest on both arms and repeated on the arm with the highest values. The average of the second and third reading was used for analysis. Hypertension was defined as systolic or diastolic blood pressure of ≥ 140/90 mmHg or use of blood pressure lowering medication.

#### Biological measurements

Total cholesterol, high-density lipoprotein (HDL) cholesterol, low- density lipoprotein (LDL) cholesterol, triglycerides, random glucose and HbA1 were measured in all individuals. For HIV-positive participants viral load and CD4 count was measured.^21^

#### Cardiovascular risk estimation

Cardiovascular disease risk was estimated using the FHS-CVD,^18^ FHS-CHD,^17^ ASCVD^20^ and HIV-specific D:A:D prediction equations.^14,28^ Details on the risk prediction equations and information regarding the different cohorts and inclusion criteria for the development of the risk scores are shown in Table 1. The cohorts for FHS and the ASCVD risk prediction models have a follow-up of over 10 years, while the D:A:D cohort has a median follow-up of five years. For the current analysis, we assumed that the CVD risk was constant over time, allowing us to predict 10-year CVD risk. From the FHS, we chose to include both the CVD and the CHD prediction model in our analysis, as these are widely used for risk estimation in clinical practice. Generally, the D:A:D 2010 algorithm is applied in clinical practice for risk estimation in PLHIV, motivating our choice to use this algorithm as reference for our comparative analysis of general CVD risk prediction scores.^29^

**TABLE 1:** Overview of the cardiovascular disease risk prediction models used in this study, showing the population characteristics and variables used by the different models.

Model	Cohort	Setting	Study population (*n*)	Age†	Follow-up† (median)	Variables (used in the model)								
						Age	G	BP	BP med	Cholesterol	DM	Smoking	HIV	cART
D:A:D, 2010^14^	D:A:D study	Europe, Australia and United States	All HIV-infected persons; Total 22 625 including 16 765 men and 5860 women, aged 16–85 years (median age = 40 years) No CVD	16–85	4.8	x	x	Sys	-	Tot/HDL	x	x	x	x
D:A:D, 2016^28^	D:A:D study	Europe and Australia	All HIV-infected participants: Total 32 663 including 24 170 men and 8493 women (median age 39 years with 10.5% non-white population) No CVD	16–85	5	x	x	Sys/dia	x	Tot/HDL	x	x	x	x
FHS-CVD, 2008^18^	Framingham heart study	Framingham,Massachusetts, United States	8.491 including 3969 men and 4522 women (median age 49 years) No CVD	30–74	12	x	x	Sys/dia	x	Tot/HDL or LDL/HDL	x	x	-	-
FHS-CHD, 1998^19^	Framingham heart study	Framingham,Massachusetts, United States	5.345 including 2489 men and 2856 women (median age 49 years) No overt CHD	30–74	> 12	x	x	Sys	-	Tot/HDL	x	x	-	-
ASCVD, 2013^20^	New pooled cohort equation	United States	Total 24 626 including 10 745 men and 13 881 women (median age 59 including 9.1% African American men and women) No CVD	40–79	> 12	x	x	Sys	-	Tot/HDL	x	x	-	-

G, gender; BP, blood pressure; BP med, using BP medication; Sys, systolic; Dia, diastolic; Tot, total; DM, diabetes mellitus; cART, combination antiretroviral therapy; HDL, high-density lipoprotein; LDL, low-density lipoprotein; CVD, cardiovascular disease; ASCVD, Atherosclerotic Cardiovascular Disease Risk Score; D:A:D, Data Collection on Adverse Events of Anti-HIV Drugs; FHS-CHD, Framingham Heart Study-coronary heart disease; FHS-CVD, Framingham Heart Study general CVD.

† , Years.

Details of the endpoints used by these different risk prediction models are described in Appendix Table 1-A1. All risk prediction models included CHD-related deaths, myocardial infarction, stroke, and stroke death as outcome, except for the FHS-CHD score, which only assessed CHD death and myocardial infarction. Details on equations used to calculate the individualised CVD risk by different CVD risk prediction tools are shown in Appendix Figure 1-A1.

### Data management and statistical analysis

Baseline characteristics of the study participants are presented as mean and standard deviation (s.d.) for normally distributed continuous variables, median and interquartile range (IQR) for non-normally distributed continuous variables and for categorical variables as count and percentage. There was a high percentage of blood pressure data obtained by a non-validated blood pressure device (61.6%) and hence discarded. Missing values on blood pressure were assumed to be missing at random (MAR). Multiple imputations were performed using the ‘MICE’ package on R, generating 15 imputed data sets on blood pressure by use of R Studio (Integrated Development Environmental for R, Inc., Boston, Massachusetts, United States).^29^ All valid blood pressure measurements, gender, age, body mass index (BMI), smoking status, glucose level, total cholesterol, HIV medication use, employment, and education were included in the imputation model. Each parameter of interest in each imputed data set was estimated separately and later combined with Rubin’s rule for analysis. Convergence plots were used to confirm that the MICE algorithm was converted. Complete case analysis was performed with the mean of the imputed data sets.

Following individual CVD risk calculation, the overall cumulative cardiovascular risk for each model was then compared using a cumulative density plot. Hence, HIV-infected study participants were categorised as having low risk (< 10%), moderate risk (10% – 20%), and high-risk (> 20%) of a CVD event within 10 years as categorised in previous similar studies.^30,31^ These categories were plotted to compare the predictions of the general CVD risk prediction models (ASCVD and FHS-CVD and FHS-CHD) with the HIV-specific D:A:D models. Furthermore, we performed subgroup analysis according to age. Since age is the most important predictor in the prediction models, it can be expected that predicted risks are higher in the older age groups.

In addition, we assessed agreement of the 2010 D:A:D risk prediction model with the other models by use of kappa statistics (0.00–0.20 poor agreement, 0.21–0.40 fair agreement, 0.41–0.60 moderate agreement, 0.61–0.80 substantial agreement, and 0.81–1.00 almost perfect agreement). Sensitivity analysis on the subset of the study population for whom measurements with the validated blood pressure device were available assessed whether agreement between the 2010 D:A:D risk prediction model with the other models differed as compared to the full case analysis based on imputed blood pressure measurement data.

Data analyses were performed using Statistical Package for Social Sciences (SPSS) version 21.0 (IBM Corp., Armonk, New York, United States).^32^ The cumulative risk graphs and CVD risk category graphs were constructed in Microsoft Office Excel (version 2010, Microsoft Corp., Redmond, Washington, United States).^33^

### Ethical considerations

Study approval was obtained from the Limpopo Department of Health Ethics Committee in Limpopo, South Africa (227/2014), and from the Human Research Ethics Committee at the University of Pretoria, Pretoria, South Africa (M160130). Written informed consent was obtained from all participants prior to study enrolment.

## Results

### Study population characteristics

The majority of the 735 participants were women (56.7%). A third (32%) of the study participants were classified as overweight and obese, and the prevalence of diabetes was 3.5%. Current smoking was reported by 23.1% of the participants. Further details on study population characteristics are provided in Table 2.

**TABLE 2:** Baseline characteristics.

Variable	Men (*N* = 318)						Women (*N* = 417)						Overall (*N* = 735)					
	*n*	%	Mean	s.d	Median	IQR	*n*	%	Mean	s.d	Median	IQR	*n*	%	mean	s.d	Median	IQR
**Age (years)**	-	-	46.1	9.2	-	-	-	-	42.2	8.2	-	-	-	-	43.9	8.8	-	-
Body mass index (BMI) kg/m^2^ M (missing values = 0)	-	-	21.2	4.3	-	-	-	-	25.4	6.3	-	-	-	-	23.6	5.9	-	-
**Income** **(missing values = 49)**																		
Low (< R648.00†/month)	161	57.7	-	-	-	-	260	63.9	-	-	-	-	421	61.4	-	-	-	-
Medium (R648.00–R992.00†/month)	13	4.7	-	-	-	-	38	9.3	-	-	-	-	51	7.4	-	-	-	-
High (> R992.00†/month)	105	37.6	-	-	-	-	109	26.8	-	-	-	-	214	31.2	-	-	-	-
**Education** **(missing values = 0)**																		
None	15	4.7	-	-	-	-	20	4.8	-	-	-	-	35	4.8	-	-	-	-
Primary	104	32.7	-	-	-	-	74	17.7	-	-	-	-	178	24.2	-	-	-	-
Secondary & matric	186	58.5	-	-	-	-	289	69.3	-	-	-	-	475	64.6	-	-	-	-
College & university	13	4.1	-	-	-	-	34	8.2	-	-	-	-	47	6.4	-	-	-	-
**Cholesterol**																		
Total cholesterol (mmol/L), (missing values = 0)	-	-	4.1	1.0	-	-	-	-	4.5	1.01	-	-	-	-	4.3	1.0	-	-
HDL cholesterol (mmol/L) (missing values = 0)	-	-	1.4	0.5	-	-	-	-	1.5	0.4	-	-	-	-	1.4	0.4	-	-
LDL cholesterol (mmol/L) (missing values = 140)	-	-	2.1	0.9	-	-	-	-	2.5	0.9	-	-	-	-	2.4	0.9	-	-
**Blood pressure**																		
Systolic blood pressure (mmHg) (missing values = 435)	-	-	116.1	14.5	-	-	-	-	114.6	17.06	-	-	-	-	115.3	16.0	-	-
Diastolic blood pressure (mmHg) (missing value = 435)	-	-	75.0	9.0	-	-	-	-	73.4	10.4	-	-	-	-	74.1	9.9	-	-
Hypertension treatment (missing values = 58)	6	1.9	-	-	-	-	20	4.8	-	-	-	-	26	3.5	-	-	-	-
Family history of CVD (missing values = 0)	43	13.5	-	-	-	-	84	20.1	-	-	-	-	127	17.3	-	-	-	-
Current smoking (missing values = 0)	136	42.8	-	-	-	-	34	8.2	-	-	-	-	170	23.1	-	-	-	-
Alcohol consumption (missing values = 0)	135	42.5	-	-	-	-	62	14.9	-	-	-	-	197	26.8	-	-	-	-
Diabetes (missing values = 0)	12	3.8	-	-	-	-	14	3.4	-	-	-	-	26	3.5	-	-	-	-
**HIV and ART status (missing values = 0)**																		
HIV-positive ART naïve	44	13.8	-	-	-	-	54	12.9	-	-	-	-	98	13.3	-	-	-	-
HIV-positive ART	274	86.2	-	-	-	-	363	87.1	-	-	-	-	637	86.7	-	-	-	-
**On cART treatment** **(missing values = 0)**	270	84.9	-	-	-	-	362	86.8	-	-	-	-	632	86.0	-	-	-	-
Abacavir	7	2.6	-	-	-	-	9	2.5	-	-	-	-	16	2.5	-	-	-	-
Lamivudine	14	5.2	-	-	-	-	31	8.6	-	-	-	-	45	7.1	-	-	-	-
Zidovudine	4	1.5	-	-	-	-	14	3.9	-	-	-	-	18	2.8	-	-	-	-
Emitricitabine	254	94.1	-	-	-	-	330	91.2	-	-	-	-	584	92.4	-	-	-	-
Tenovofir	256	94.8	-	-	-	-	316	87.3	-	-	-	-	572	90.5	-	-	-	-
**Last CD4+ cell count,** cells/mm^3^ (missing values = 8)	-	-	-	-	404.0	257–583	-	-	-	-	513	369–707	-	-	-	-	469	312–656
Last CD4+ cell count, 200 cells/mm^3^ – 350 cells/mm^3^	77	24.2	-	-	-	-	63	15.1	-	-	-	-	140	19.0	-	-	-	-
Last CD4+ cell count, > 350 cells/mm^3^	187	58.8	-	-	-	-	315	75.5	-	-	-	-	502	68.3	-	-	-	-
**Viral load,** copies/mL, per log 10 (missing values = 458)	-	-	-	-	1677.50	82–28 738	-	-	-	-	2331	112–12 924	-	-	-	-	1763	87–16 517
Viral load (missing values = 7)																		
< 50 copies/mL	198	62.3	-	-	-	-	298	71.5	-	-	-	-	496	67.5	-	-	-	-
50–1000 copies/mL	38	11.9	-	-	-	-	37	8.9	-	-	-	-	75	10.2	-	-	-	-
> 1000 copies/mL	80	25.2	-	-	-	-	77	18.5	-	-	-	-	157	21.4	-	-	-	-

s.d., standard deviation; IQR, interquartile range; CVD, cardiovascular disease; HDL, high-density lipoprotein; LDL, low-density lipoprotein; ART, antiretroviral treatment; cART, combined antiretroviral treatment.

† , South African rand (ZAR).

### Cardiovascular disease risk estimation

**FIGURE 2:**
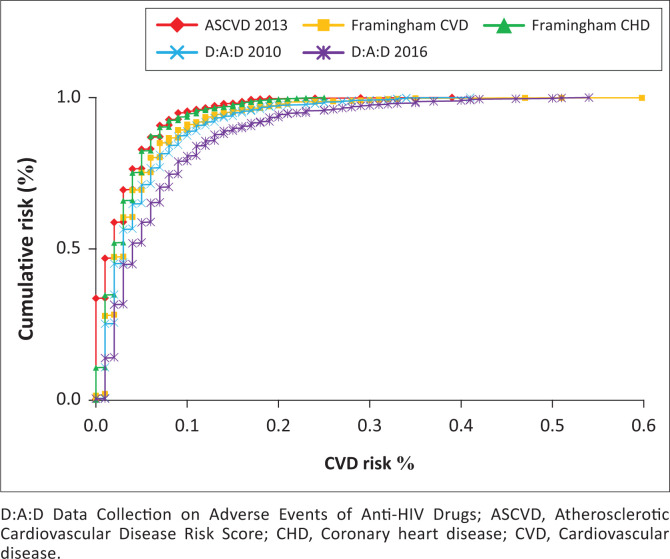
Cumulative risk for all Ndlovu Cohort Study HIV-positive study participants for the cardiovascular disease risk prediction models.

The D:A:D 2010 and Framingham CVD risk prediction models gave similar cumulative risk distributions. The cumulative predicted 10-year CVD risk between D:A:D 2010 and FHS-CVD was 4% and for ASCVD and FHS-CHD, 3%. For D:A:D 2016, the figure was 5% (Figure 2). While median predicted 10-year CVD for FHS-CVD was 3% (IQR = 1% – 3%), figures for FHS-CHD were 2% (IQR = 1% – 4%), 2% for ASCVD (IQR = 1% – 4%), 3% for D:A:D 2010 (IQR = 1% – 6%) and 7% for D:A:D 2016 (IQR = 3% – 14%).

**FIGURE 3:**
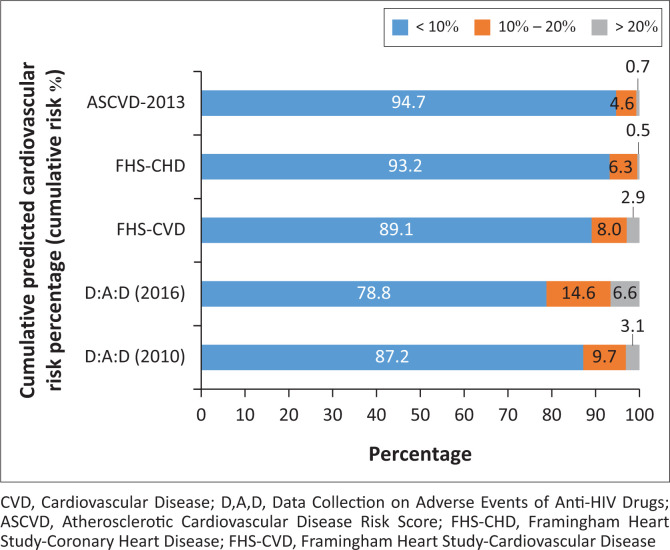
Cardiovascular disease risk categories. The 10-year cardiovascular disease risk is depicted in three categories, low (< 10%), medium (10% – 20%), and high (> 20%).

For FHS, ASCVD and D:A:D 2010, a similar distribution of participants in the low-risk (> 80%), moderate-risk (5% – 10%), and high-risk (1% – 3%) categories was observed; for D:A:D 2016, less than 80% were classified at low risk, 15% at moderate risk and > 6% as high risk for CVD (Figure 3).

In subgroup analysis, we analysed CVD risk within different age groups. For the age category 30–39-years, all were categorised as low risk by all the prediction models; for the age category 40–49-years, 97.0% were categorised as low risk, 3.0% as moderate risk, and ≤ 1.0% as high risk. For those aged 50–59-years, 68.3% were categorised as low risk, 25.4% as moderate risk, and 6.3% as higher risk; for those aged 60 years or older, 30.0% were categorised as low risk, 42.0% as moderate risk, and 28.0% as higher risk.

The FHS-CVD, FHS-CHD and ASCVD classified 91.4, 89.3 and 88.8% in the same category as the D:A:D 2010 prediction model. Agreement between D:A:D 2010 and 2016 was 87.0%. In a subgroup analysis of participants aged 40 years and older, a lower agreement was observed: 88.0% between D:A:D 2010 and FHS-CVD, and 83.2 and 82.5% for FHS-CHD and ASCVD. The highest predicted CVD risk and disagreement was observed for D:A:D 2016 when compared with the D:A:D 2010 risk prediction model.

The weighted kappa statistic ranged from 0.34 for ASCVD (*P* < 0.001) to 0.60 for FHS-CVD (*P* < 0.001) compared to 2010 D:A:D. Further details are shown in Table 3.

**TABLE 3:** Risk prediction: comparing the general cardiovascular disease models with the HIV-specific data collection on adverse events of anti-HIV drugs (D:A:D) 2010 model.

D:A:D 2010	FHS (CVD)			FHS (CHD)			ASCVD			D:A:D 2016			Total	Kappa statistics results	*p*
	Low (< 10%)	Medium (10% – 20%)	High (> 20%)	Low (< 10%)	Medium (10% – 20%)	High (> 20%)	Low (< 10%)	Medium (10% – 20%)	High (> 20%)	Low (< 10%)	Medium (10% – 20%)	High (> 20%)			
**D:A:D 2010**															
< 10%	621	19	1	-	-	-	-	-	-	-	-	-	641	Agreement: 91.4% Kappa statistics: 0.60	< 000.1
10% – 20%	30	36	5	-	-	-	-	-	-	-	-	-	71		
> 20%	4	4	15	-	-	-	-	-	-	-	-	-	23		
Total	655	59	21	-	-	-	-	-	-	-	-	-	735		
**D:A:D 2010**															
< 10%	-	-	-	631	10	0	-	-	-	-	-	-	641	Agreement: 89.3% Kappa statistic: 0.41	< 000.1
10% – 20%	-	-	-	50	21	0	-	-	-	-	-	-	71		
> 20%	-	-	-	4	15	4	-	-	-	-	-	-	23		
Total	-	-	-	685	46	4	-	-	-	-	-	-	735		
**D:A:D 2010**															
< 10%	-	-	-	-	-	-	637	3	1	-	-	-	641	Agreement: 88.8% Kappa statistic: 0.34	< 000.1
10% – 20%	-	-	-	-	-	-	57	13	1	-	-	-	71		
> 20%	-	-	-	-	-	-	2	18	3	-	-	-	23		
Total	-	-	-	-	-	-	696	34	5	-	-	-	735		
**D:A:D 2010**															
< 10%	-	-	-	-	-	-	-	-	-	-	568	63	2	Agreement: 87.0% Kappa statistic: 0.56	< 000.1
10% – 20%	-	-	-	-	-	-	-	-	-	-	4	42	25		
> 20%	-	-	-	-	-	-	-	-	-	-	0	0	21		
Total	-	-	-	-	-	-	-	-	-	-	572	105	48		

Note: Patients were divided into three categories: < 10%, 10% – 20% and > 20% risk of CVD. ‘Agreement’ indicates the degree to which the model categorises the patients in the same way as the D:A:D 2010 model. The kappa statistics are included.

CVD, cardiovascular disease; FHS, Framingham heart study; CHD, coronary heart disease; ASCVD, atherosclerotic cardiovascular disease.

Sensitivity analysis showed overall comparable results for the degree of agreement between the 2010 D:A:D and the other prediction models (FHS-CVD agreement 90%, kappa 0.46; FHS-CHD 91%, 0.43; ASCVD 91%, 0.34; D:A:D 2016 74%, 0.60).

## Discussion

We showed that FHS-CVD and D:A:D 2010 risk equations predict relatively similar 10-year CVD and 5-year CVD risk in PLHIV in a rural community in South Africa. Comparison of FSH-CHD and ASCVD with D:A:D 2010 risk classification showed a similar distribution of participants over the risk groups. Compared to D:A:D 2010, the D:A:D 2016 risk prediction model showed overall good agreement but predicted relatively fewer participants in the low-risk category and more in the high-risk CVD risk category. The results indicate that all general CVD risk prediction models applied in the current study classified a similar percentage of PLHIV to be at low CVD risk.

The ASCVD and FHS-CHD categorised few people to be at high risk of CVD compared to D:A:D 2010 and FHS-CVD, illustrating that use of the ASCVD and FHS-CHD models could lead to a lower CVD risk estimation. On the other hand, D:A:D 2016 categorised more people at high CVD risk compared to D:A:D 2010, highlighting that the use of the D:A:D 2016 algorithm could lead to higher CVD risk estimation. Comparison of the HIV-specific D:A:D 2010 to the recalibrated D:A:D 2016 algorithm showed a higher percentage of PLHIV at the NCS to be classified at high CVD risk in the 2016 model. The possible reasons for these higher percentage by D:A:D 2016 model might be that the model includes the full effect of protease inhibitors on CVD, including their lipids and independent drug effect and CD4 lymphocyte cell count. Furthermore, D:A:D 2016 includes endpoints such as heart failure, not included in D:A:D 2010, which could also lead to higher estimates. Moreover, it is considered that ethnic or racial status is a significant predictor of CVD risk.^34^ To date, however, most CVD prediction models^35^ are not taking into consideration ethnic or racial status, which could be a possible reason for the lower estimates observed in the NCS, a study undertaken in a Black African population in rural SSA. Ethnic or racial status should be considered as a potentially important factor when developing prediction models. Currently it is not known what risk prediction model more accurately estimates CVD risk in a SSA setting.

A previous study in PLHIV in Portugal,^31^ showed high CVD risk with the FHS-CVD and FHS-CHD models, than by the 2010 D:A:D, ASCVD, and Systematic Coronary Risk Evaluation for the Netherlands (SCORE-NL) models. In contrast to these results, in the current study, comparable risk prediction was observed for D:A:D 2010, FHS-CVD, FHS-CHD, and ASCVD for those at low CVD risk. For those at high CVD risk, D:A:D 2010 and FHS-CVD provided similar results, whereas the 2016 D:A:D model classified a higher percentage of PLHIV at the NCS to be at high CVD risk.

Overall, in the current study, few participants were categorised to be at high CVD risk. This is most likely related to the young age of the study population and the relatively low prevalence of traditional CVD risk factors, such as smoking, abnormal lipid levels and alcohol consumption. Conventional CVD risk factors have been shown in several studies to be lower for PLHIV compared to the general population in low- and middle-income settings.^7,26,36,37^ Underlying reasons for this observation, however, remain unclear.^37,38^ One possibility is increased access to healthcare and regular health checks for PLHIV.^39^

There are numerous risk prediction algorithms to calculate CVD risk.^40,41,42^ The FHS-CVD, FHS-CHD and ASCVD are among the most frequently used and well-known CVD risk algorithms. These prediction models are recommended by many guidelines for the prevention and management of CVD.^43^ Our study shows that these risk prediction models classified PLHIV at the NCS to be at similarly low, medium or high CVD risk as the HIV-specific D:A:D 2010 model. These risk algorithms are all based on cohorts with a long follow-up and are generally available in the form of easy-to-use electronic calculators.^45,46^

The results of our study show that all models predicted relatively similarly in the low-risk category; therefore, over-estimation in a low-risk population is unlikely by use of any of the risk prediction models. For PLHIV with a moderate and high risk of CVD under-treatment, and for those with a low risk, over-treatment could be avoided, potentially resulting in drug-related adverse reactions, drug resistance, and drug-drug interactions. Risk stratification could guide preventive measures and efficient use of resources until a CVD SSA prediction model has been developed and validated.

To identify the CVD risk prediction models most applicable to PLHIV in the general population in different settings over time, longitudinal studies collecting clinical outcome data are recommended. The D:A:D model has been calibrated for a 5-year risk prediction. Therefore, extrapolation is required to predict a 10-year CVD risk. The assumption in this study is that risk is stable over time, thus extrapolation of the estimates of the D:A:D models leads to greater uncertainty and a higher risk of producing less relevant results. This study is one of few studies that have added the recalibrated D:A:D 2016 risk prediction model in their analysis to provide a better picture of estimates and results in SSA populations.

In conclusion, CVD risk estimation using Framingham CVD as compared to the HIV-specific D:A:D 2010 model showed similar results for risk classification of PLHIV. This study demonstrates that currently used CVD risk prediction models show good agreement in the low CVD risk category, whereas the observed agreement is moderate for the medium-risk and high-risk categories. To enable the development or validation of CVD risk algorithms that can be used broadly in PLHIV in SSA populations, prospective studies are recommended. Information on newer antiretrovirals^44^ along with other CVD risk factors, and clinical outcomes,^7,26,47^ could lead to refined CVD prediction models for use in the SSA setting.^48^

## Figures and Tables

**FIGURE 1-A1:**
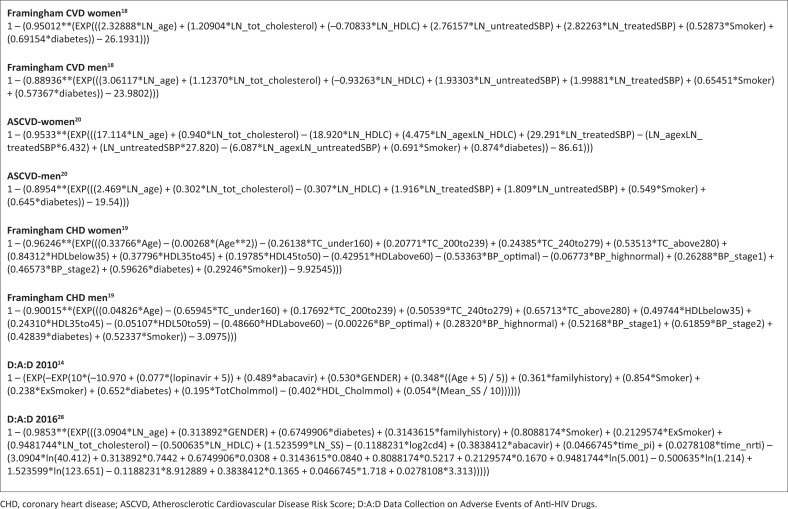
Equations used to calculate individualised cardiovascular disease.

## Appendix 1

**TABLE 1-A1:** Endpoint definitions used by the cardiovascular disease risk prediction models.

Model	Cor. insuf. or revasc.	Angina	Unstable angina	AMI	CHD death	Stroke	Stroke death	Cardiac failure	TIA	PAD
D:A:D 2010^14^	x	x	x	x	x	x	x	-	-	-
D:A:D 2016^28^	x	x	x	x	x	x	x	x	-	-
FHS-CVD^18^	x	x	-	x	x	x	x	x	x	x
FHS-CHD^19^	x	x	x	x	x	-	-	-	-	-
ASCVD^20^	-	-	-	x	x	x	x	x	-	-

CHD, coronary heart disease; Cor. insuf. or revasc., coronary insufficiency or revascularisation; AMI, acute myocardial infarction; TIA, transient ischaemic attack; PAD, peripheral artery disease; ASCVD, Atherosclerotic Cardiovascular Disease Risk Score; D:A:D Data Collection on Adverse Events of Anti-HIV Drugs; FHS-CHD, Framingham Heart Study-coronary heart disease; FHS-CVD, Framingham Heart Study-cardiovascular disease; revasc., revascularisation.
